# Transient genome-wide interactions of the master transcription factor NLP7 initiate a rapid nitrogen-response cascade

**DOI:** 10.1038/s41467-020-14979-6

**Published:** 2020-03-02

**Authors:** José M. Alvarez, Anna-Lena Schinke, Matthew D. Brooks, Angelo Pasquino, Lauriebeth Leonelli, Kranthi Varala, Alaeddine Safi, Gabriel Krouk, Anne Krapp, Gloria M. Coruzzi

**Affiliations:** 1 Center for Genomics and Systems Biology, New York University, New York, NY USA; 2 Centro de Genómica y Bioinformática, Facultad de Ciencias, Universidad Mayor, Santiago, Chile; 3 Department of Horticulture and Landscape Architecture, Purdue University, West Lafayette, IN USA; 4 BPMP, Université de Montpellier, CNRS, INRA, SupAgro, Montpellier, France; 5 Institut Jean-Pierre Bourgin, INRAE, AgroParisTech, Université Paris-Saclay, 78000 Versailles, France

**Keywords:** Gene expression analysis, Plant molecular biology, Plant signalling, Dynamic networks

## Abstract

Dynamic reprogramming of gene regulatory networks (GRNs) enables organisms to rapidly respond to environmental perturbation. However, the underlying transient interactions between transcription factors (TFs) and genome-wide targets typically elude biochemical detection. Here, we capture both stable and transient TF-target interactions genome-wide within minutes after controlled TF nuclear import using time-series chromatin immunoprecipitation (ChIP-seq) and/or DNA adenine methyltransferase identification (DamID-seq). The transient TF-target interactions captured uncover the early mode-of-action of NIN-LIKE PROTEIN 7 (NLP7), a master regulator of the nitrogen signaling pathway in plants. These transient NLP7 targets captured in root cells using temporal TF perturbation account for 50% of NLP7-regulated genes not detectably bound by NLP7 in planta. Rapid and transient NLP7 binding activates early nitrogen response TFs, which we validate to amplify the NLP7-initiated transcriptional cascade. Our approaches to capture transient TF-target interactions genome-wide can be applied to validate dynamic GRN models for any pathway or organism of interest.

## Abstract (web summary)

Conventional methods cannot reveal transient transcription factors (TFs) and targets interactions. Here, Alvarez *et al*. capture both stable and transient TF-target interactions by time-series ChIP-seq and/or DamID-seq in a cell-based TF perturbation system and show NLP7 as a master TF to initiate a rapid nitrogen-response cascade.

## Introduction

Dynamic interactions of regulatory proteins with DNA are important to trigger temporal responses to a changing cellular or external environment. However, transient interactions between transcription factors (TFs) and their genome-wide targets are largely missed in validated gene-regulatory networks (GRNs). This is because across eukaryotes, TFs are detectably bound to only a small percentage of their regulated targets, as shown in plants^1–3^, yeast^4^, and animals^5,6^. Paradoxically, the very large set of TF-regulated, but unbound genes are typically dismissed as indirect targets, because standard approaches can only identify direct TF targets based on TF binding^7,8^. The alternative hypothesis is that the TF-regulated, but unbound genes are in fact direct targets that are only transiently bound by the TF. However, capturing short-lived or transient TF-target binding by standard biochemical methods such as chromatin immunoprecipitation (ChIP) is difficult^7,9,10^ due to (i) physical instability, and (ii) the limited number and frequency of the time points that can be analyzed. This problem is exacerbated in multicellular eukaryotes where the time required to fix TF–DNA complexes in whole tissues (~20 min) is magnitudes longer than signal propagation within cells (seconds to minutes)^7^.

The master TF regulator NIN-LIKE PROTEIN 7 (NLP7), which controls very early nitrogen (N) responses in *Arabidopsis thaliana*, is a prime example of the disconnect between TF binding and regulation^11–13^. Paradoxically, while NLP7 nuclear localization by phosphorylation occurs within minutes of N supply, the vast proportion of genes regulated by NLP7 in planta (~90%) are not detectably bound in ChIP-chip assays^11,13^. These elusive NLP7 targets may involve transient NLP7 interactions that defy biochemical detection (e.g., by ChIP), and/or indirect control through NLP7-regulated transcription factors that trigger a broad transcriptional cascade^11,12^.

To capture potential transient TF–target interactions of NLP7, we exploited the cell-based TARGET system, which can identify direct TF-mediated gene regulation: (i) in the absence of stable TF binding, and (ii) within minutes of controlled TF nuclear entry, using time-series ChIP^14,15^. We previously used this system to capture early and transient TF–target interactions for bZIP1, which support a Hit-and-Run model of transcription, in which transient TF binding initiates transcription that persists long after the TF has dissociated from its targets^14,16^. However, ChIP—a biochemical assay—is only a snapshot of the most stable TF-binding events under the conditions and time points assayed^9,10^. Thus, herein we employed a DNA adenine methyltransferase (Dam)–TF fusion protein to mark (by DNA methylation) promoters touched, even transiently, by the TF (DamID)^17^. The stable adenine methylation signature left on the DNA, allows one to capture even the briefest TF–DNA interaction, a major advantage of DamID over ChIP (reviewed in Aughey and Southall^17^).

Herein, we capture three classes of NLP7 targets genome wide: stable, transient, and highly transient using a combination time-series ChIP and DamID in root cells using the TARGET system. Importantly, the NLP7 transient and highly transient targets we captured in roots cells account for 50% of the NLP7 regulated, but unbound genes, in planta^11^. Moreover, these transient targets of NLP7 are enriched in early N-responsive TFs, including ones validated in planta—LBD37 & LBD38^18^, CDF1^19^, TGA4^20,21^—but not previously known to act downstream of NLP7. Our validation studies of these and other secondary TFs—HAP2C, NAC096, and Integrase-type DNA (At4g39780)—acting downstream of NLP7 show they regulate 53% of the N-responsive genes in whole roots. Thus, our studies uncover the path by which NLP7 regulates dynamic and early N responses, and where it fits in the temporal hierarchy of the N transcriptional network.

## Results

### Capturing direct NLP7 targets by TF regulation or TF binding

**Fig. 1:**
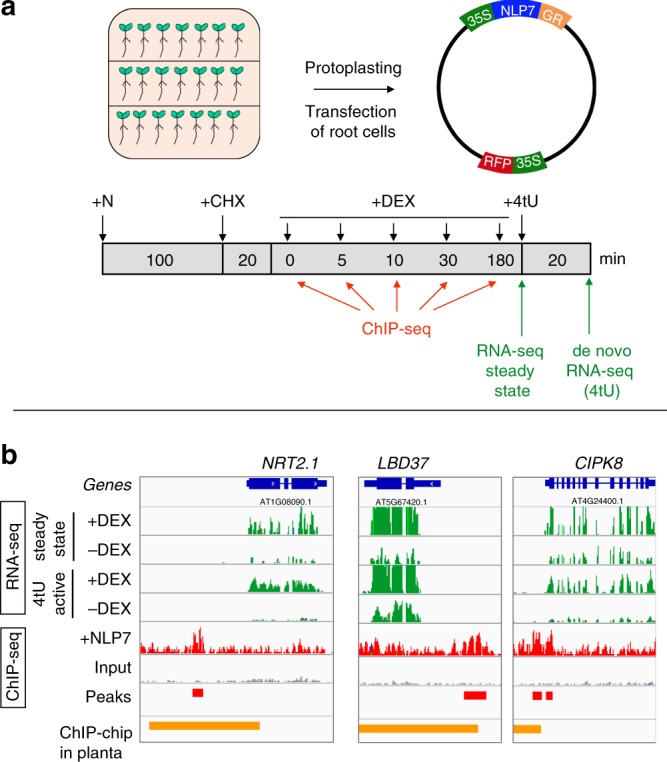
TARGET TF-perturbation assay captures direct targets of NLP7 based on TF regulation or TF binding in isolated root cells. **a** Schematic of the cell-based TARGET TF-perturbation system and experimental design^22^ (see “Methods”). Root cells isolated from an *nlp7* mutant^11,23^ transfected with a 35S::NLP7-GR construct were allowed to express the TF–GR fusion protein and sequentially treated with (i) the nitrogen (N) signal transduced by the TF, (ii) cycloheximide (CHX) to block translation, allowing mRNA synthesis of only direct NLP7 targets, (iii) dexamethasone (DEX) to induce NLP7-GR nuclear import. Samples for NLP7 binding to targets as assayed by ChIP using anti-GR antibodies were collected after 0, 5, 10, 30, and 180 min of DEX-induced TF nuclear import. Genes whose expression is affected NLP7 nuclear import were assayed by RNA-seq (steady-state mRNA) or by affinity capture of de novo mRNA using 4tU^16^. **b** Representative examples of direct targets transcriptionally activated by NLP7. The expression of *NRT2.1*, *LBD37*, and *CIPK8* is induced by +DEX as compared with −DEX treatment in both steady state and 4tU-enriched RNA-seq experiments (green bars). NLP7 binding was captured by ChIP-seq, and NLP7 peaks were identified by MACS2 using an input sample as a control (red bars) (see “Methods”). NLP7 peaks identified in whole roots by ChIP-chip (orange bars)^11^.

Here, we set out to address the paradox that NLP7 binds to hundreds of genes, yet only 10% of NLP7-regulated targets are NLP7-bound based on ChIP-chip assay in planta^11^. To test the hypothesis that NLP7 binds transiently to a subset of its targets, we used the root cell-based TARGET system for temporal TF perturbation^22^, which can capture TF binding by ChIP within 1 min of controlled TF nuclear entry^14^. Using the TARGET assay, we transiently expressed an NLP7-glucocorticoid receptor (GR) fusion protein (NLP7-GR) in cells isolated from whole roots of the *Arabidopsis nlp7* mutant^11,23^. Briefly, following vector transfection of the NLP7-GR construct into isolated root cells, we induced nuclear import of the NLP7-GR fusion protein using dexamethasone (DEX) treatment (Fig. 1a). As N treatment has previously been shown to promote nuclear retention of NLP7^11^, we also examined the effects of N (NO_3_^−^ and NH_4_^+^) or DEX, alone and in combination. To do this, we quantified NLP7 activation of a reporter YFP gene driven by the nitrate responsive promoter (NRP) (Supplementary Fig. 1), which NLP7 has been shown to bind to and regulate in roots of whole plants^11,24^. These results showed a synergistic effect of DEX (Supplementary Fig. 1), which promotes TF–GR nuclear entry^25,26^ and N treatment that promotes nuclear retention of NLP7^11,13^ on gene induction (Supplementary Fig. 1). Thus, we performed sequential treatment with both N and DEX to induce NLP7 nuclear import in root cells of *nlp7* mutants transfected with the NLP7-GR construct (Fig. 1a). Induction of NLP7-GR nuclear entry by DEX-treatments was performed in the presence of cycloheximide (CHX) to prevent translation of downstream regulators, as shown previously in the cell-based TARGET system^14,21,22^ and also in planta^25,26^. The combined +DEX/+CHX treatments allowed us to identify direct regulated NLP7 targets using RNA-seq. We also identified de novo mRNAs resulting from NLP7 nuclear import using 4tU-affinity labeling and capture of nascent transcripts^16,27^ (Fig. 1a).

Using these approaches, we could identify direct NLP7-regulated targets by significant changes in their steady-state levels of mRNA (RNA-seq, steady state) and/or by affinity capture of de novo mRNA (RNA-seq, 4tU), as in ref. ^16^. Examples are shown for three NLP7 targets also previously validated in planta^11^: *NITRATE TRANSPORTER 2.1* (*NRT2.1*), *LATERAL ORGAN BOUNDARY DOMAIN 37* (*LDB37*), and *CBL-INTERACTING PROTEIN KINASE 8* (*CIPK8*), which are involved in N transport^28,29^, transcriptional regulation^18^, and N-signaling^30^, respectively (Fig. 1b, green bars). *NRT2.1*, *LDB37*, and *CIPK8* are direct regulated targets of NLP7, as RNA-seq data revealed their expression was highly induced by NLP7 nuclear import (+DEX) under +CHX treatments (Fig. 1b, green bars). In addition, these genes are actively transcribed in response to NLP7 controlled nuclear import, as shown 4tU-affinity labeling and capture of de novo mRNA made following NLP7 nuclear import (Fig. 1b, green bars). To capture NLP7-target binding, we used anti-GR antibodies against the NLP7-GR fusion protein to perform ChIP-seq (Fig. 1a). The resultant ChIP-seq peaks for the three genes described above are shown in Fig. 1b (red bars). These results show that the NLP7-binding sites identified by ChIP-seq in root cells using the TARGET assay, overlap with NLP7-binding sites previously identified by ChIP-chip in planta in the promoters of *NRT2.1*, *LDB37*, and *CIPK8* (Fig. 1b, orange bars)^11^. These results demonstrate that the cell-based TARGET system can identify direct NLP7 targets by mRNA analysis or by TF–DNA binding in root cells that are bona fide direct NLP7 targets shown in planta^11^.

### NLP7-binding sites are maximal minutes after nuclear import

Next, to capture the dynamics of NLP7-target binding genome wide, we performed a time-series ChIP-seq experiment at 5, 10, 30, and 180 min after DEX-induced import of the NLP7-GR protein into nuclei of root cells isolated from the *nlp7* mutant (Fig. 1a). The control was time 0 before DEX-induced TF nuclear import. We compared NLP7-target binding levels across time points by calculating reads per kilobase per million reads (RPKM) in each sample. Genomic locations bound by NLP7 were compared in parallel across all time points in these normalized samples (Supplementary Fig. 2a).

This time-series ChIP-seq analysis showed that genome-wide binding of NLP7 to its targets was highest at 5 min following TF nuclear import, compared with time 0 or later time points (Supplementary Fig. 2a, b). Low levels of NLP7 binding were detected at time 0 (Supplementary Fig. 2a, b), hence time 0 was excluded from further analyses. In addition, we found that a high proportion of NLP7 peaks are located close to the transcription start site (TSS) of genes in root cells (Supplementary Fig. 3a), as previously described for NLP7 in whole roots^11^. Our time-series ChIP-seq analysis in root cells identified NLP7-target genes bound at 5 min (6288), 10 min (1299), 30 min (1518), and 180 min (861) after controlled TF nuclear import (Supplementary Data 1–4). These NLP7-bound genes captured in our time-series ChIP in root cells include a high and significant proportion (57%, *p*-value 1.6E-56, Fisher’s exact test) of previously described NLP7-bound targets in planta (Supplementary Fig. 3b)^11^. In addition, genes bound by NLP7 in root cells significantly overlapped with N-responsive genes in a time-series N-response transcriptome series data from whole roots^19^, as well as with genes regulated directly by nitrate in whole roots^31^ (Supplementary Fig. 3b). These results collectively support that NLP7-bound targets identified using the TARGET assay in root cells are enriched in bona fide NLP7 targets with relevance to the N response in planta.

### NLP7-bound targets include stable vs. transient targets

A total of 492 genes were identified as direct NLP7 targets based on gene regulation in isolated root cells using the TARGET system (Supplementary Data 5). These direct regulated NLP7 targets included genes involved in N-uptake/metabolism such as the *NITRATE TRANSPORTER 1.1* (*NRT1.1*), *NITRATE REDUCTASE 1* (*NIA1)*, *NITRATE REDUCTASE 2 (NIA2)*, and *NITRITE REDUCTASE*(*NIR)* (Supplementary Fig. 4a). In addition, the direct regulated targets of NLP7 we identified in root cells using TARGET were significantly enriched in gene ontology (GO) terms related to transcription factor regulation and predicted kinase activity (Supplementary Fig. 4b). Specifically, 62 TFs (referred to as secondary TFs) were direct regulated targets of NLP7, including known TFs involved in the N response in planta such as LBD37, LBD38, CDF1, and TGA4^18–20^ (Supplementary Fig. 4a, Supplementary Data 6). In addition, 66 proteins with kinase activity were direct regulated targets of NLP7 (Supplementary Data 7).

**Fig. 2:**
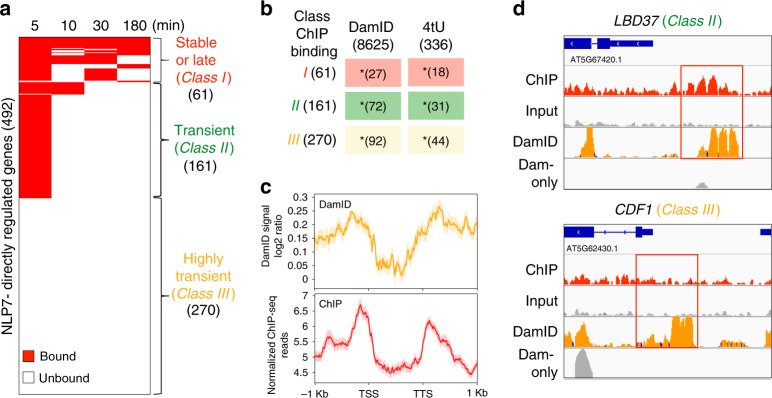
Transient and highly transiently bound NLP7 actively transcribed targets are captured by time-series ChIP and/or DamID. **a** Intersection of NLP7 directly regulated genes and NLP7-bound genes captured by time-series ChIP-seq (minutes after DEX-induced nuclear import). Red bars indicate genes that are bound and directly regulated by NLP7. Intersection of these datasets revealed three distinct classes of direct NLP7-regulated targets: (Class I) stable or late, (Class II) transient, (Class III) highly transient. **b** DamID captures a significant proportion of genes belonging to all three classes, including NLP7–target interactions that were missed by ChIP (Class III). Genes from all three classes are enriched in genes whose active transcription is induced by NLP7 (4tU-labeled). Fisher’s exact test *p-value < 0.001. **c** The profile of DamID, indicated by the number of normalized DamID sequencing reads in the 1000-bp upstream regions of TSS to the 1000-bp downstream regions of TTS. The DamID profile is similar to the ChIP profile, indicated by the normalized ChIP-seq reads. **d** *LBD37* is an example of active NLP7-initiated transcription (4tU) where binding is captured by both DamID (orange) and ChIP (red). CDF1 is an example of active NLP7-initiated transcription in which TF binding is captured by DamID, but missed by ChIP.

We identified three distinct classes of NLP7-direct target genes based on the intersection of the NLP7 ChIP-seq time-series TF-binding data and the 492 NLP7-direct regulated targets. Class I stable or late NLP7-direct targets (61 genes) were TF-bound across two or more time points, including late time points (Fig. 2a; Supplementary Data 8). Class II transient NLP7-direct targets (161 genes) were TF-bound as early as 5 min or 5 & 10 min after TF nuclear import, but TF binding was not detected at later time points (e.g., 30 and 180 min) (Supplementary Data 9). Class III highly transient NLP7 targets were direct regulated targets based on gene regulation in response to NLP7 nuclear import in the presence of +CHX. However, binding of NPL7 was not detected by ChIP at any time point tested (270 genes) (Supplementary Data 10). We hypothesized that the lack of detectable TF binding for Class III targets—the largest class of NLP7 targets—represented highly transient interactions of NLP7 that were not captured due to technical limitations of biochemical assay such as ChIP.

### Highly transient targets of NLP7 are captured using DamID

We set out to test the hypothesis that Class III highly transient direct regulated targets of NLP7 are due to short-lived interactions of the TF with a set of genome-wide targets. To do this, we adapted the DNA adenine methyltransferase IDentification (DamID) method^32^ to fingerprint NLP7-binding sites in the cell-based TARGET system. Our innovation of the DamID method is the incorporation of a GR-tag, enabling inducible nuclear localization of the Dam-GR-TF fusion protein. The stable adenine methylation mark of targets touched by the TF in vivo, allowed us to capture even the briefest TF–DNA interaction^17,32^. Our DamID analysis identified 8625 target genes that were touched by the Dam-GR-NLP7 fusion protein in root cells. Using these DamID results, we determined that NLP7 bound to 39% of its direct regulated targets (192/492) from all three classes defined by our time-course ChIP assays; stable, transient, and highly transient (Fig. 2b; Supplementary Fig. 5; Supplementary Data 11 and Supplementary Data 12). These NLP7-bound genes identified by DamID also significantly overlapped with NLP7-bound targets identified by ChIP-Chip in planta^11^, as well as with NLP7-bound targets identified by ChIP-seq in root cells (Supplementary Fig. 5). DamID enabled us to identify NLP7 binding to 34% (92/270) of the Class III highly transient directly regulated targets of NLP7, completely missed even by time-series ChIP detection (Fig. 2b, Supplementary Data 13). Although our time-series ChIP-seq captured 45% of NLP7-direct regulated targets (222/492) (Fig. 2a), genes from the Class III highly transient category of NLP7 targets captured by DamID were missed using ChIP-seq only.

Encouragingly, NLP7-target binding profiles identified using DamID-seq and ChIP-seq in root cells were strikingly similar, with both techniques showing NLP7 binding concentrated close to the transcription start site (TSS) and transcription termination site (TTS) (Fig. 2c). Sample DamID-seq data for NLP7 binding to two secondary TFs—a Class II transient target, *LBD37*, and a Class III highly transient target, *CDF1*—are shown in Fig. 2d. NLP7-binding domains detected by DamID and by ChIP-seq overlap in the promoter of the transient NLP7 target *LBD37* (Fig. 2d). For the highly transient NLP7 target *CDF1*, TF-target binding was captured only by DamID, but missed by time-series ChIP (Fig. 2d). These results demonstrate that DamID can capture highly transient targets of NLP7 that were missed even by time-series ChIP.

Although previous studies show that NLP7 regulates a myriad of N-responsive genes in roots of whole plants, a large proportion (~90%) of these regulated genes are not detectably bound in ChIP-chip assays in planta^11^. Strikingly, we found that by combining time-series ChIP and DamID targets identified in root cells using the TARGET system, we could capture 49% of the NLP7-regulated but unbound genes in planta (209 out of 430 genes, *p*-value 0.004, Fisher’s exact test) (Supplementary Fig. 6, Supplementary Data 14)^11^. These results support our hypothesis that the vast majority of transient NLP7–target interactions that result in gene regulation are missed by in planta studies. The promoters of NLP7-bound genes we captured by either ChIP or DamID in root cells were significantly enriched in the known NLP7-binding site^33^ (4424/8625 gene promoters for DamID, *p*-value 2E-17, Supplementary Data 15; 3291/6423 gene promoters for ChIP, *p*-value 5.61E-4, Fisher’s exact test, Supplementary Data 16), further supporting that they are direct targets of NLP7.

### Active transcription continues after NLP7 transient binding

We next asked whether the transient and highly transient NLP7 targets (Class II and III) are actively transcribed at times when NLP7 is no longer bound. To do this, we used 4tU-affinity labeling to capture de novo mRNA transcripts made after NLP7 nuclear import (as described by Doidy)^16^. This approach allowed us to identify NLP7 targets (336 genes) whose mRNA was actively synthesized 3 h after conditional NLP7 nuclear import (Supplementary Data 17). Active transcriptional regulation by NLP7 can largely explain changes in target gene transcript levels, as the magnitude of de novo mRNA synthesis in response to NLP7 nuclear import correlated with the magnitude of steady-state mRNA levels for this set of genes (R^2^ = 0.83) (Supplementary Fig. 7). In addition, there was a significant enrichment of de novo transcription in all three classes of NLP7-regulated direct targets (Fig. 2b). Interestingly, we found that gene targets in contact with NLP7 only transiently (e.g., at 5 min, Class II) or highly transiently (e.g., Class III NLP7 targets captured only by DamID) were still actively transcribed at 3 h, a time at which NLP7 binding was not detected by time-series ChIP (Fig. 2b).

These results show that the master TF NLP7 can regulate its genome-wide targets in one of three modes-of-action—stable, transient, or highly transient binding, depending on the target gene. We thus examined whether the cis-context could explain these different classes of genome-wide NLP7-direct targets. To do this, we performed cis-motif analysis in the promoter of the three classes of NLP7-regulated direct targets. We note that both induced (A) or repressed (B) genes from the three NLP7-target classes are enriched in genes whose transcription is regulated by NLP7 nuclear import, as captured by 4tU-affinity labeling (Supplementary Fig. 8a, b). For NLP7-induced targets (Classes IA, IIA, IIIA), each class was significantly enriched in NLP7 cis-motif identified by in vitro NLP7-DNA binding by DAP-seq^33^. In Class IA (stably bound NLP7 targets), we found no enrichment for additional cis-motifs. This result suggests that stable and direct binding NLP7 to its known cis-motif is sufficient for activation and sustained transcription (captured by 4tU labeling) of Class IA stably bound targets. In contrast, both Class IIA (transient), and Class IIIA (highly transient) targets of NLP7 are specifically enriched not only in the known NLP7 cis-element but also in cis-elements for additional TFs, including the ERF and GATA families, respectively (Supplementary Fig. 8c). These results suggest that transient NLP7 binding leads to sustained transcription, potentially through TF partners whose binding sites are specifically enriched in the transient and highly transient NLP7-induced targets (Supplementary Fig. 8c). It is noteworthy that NLP7 repressed genes are not enriched in the NLP7-binding site, even though they are bound by NLP7 (e.g., by ChIP or DamID), which suggest indirect TF–DNA binding. Instead, targets from all three classes of NLP7 repressed genes (Class IB, IIB, and IIIB) were each enriched in the W-box element (Supplementary Fig. 8c). This finding suggests NLP7 could operate through partner TF binding that recognizes the W-box element on DNA to repress gene targets.

### Transient NLP7 targets are early N-responsive secondary TFs

**Fig. 3:**
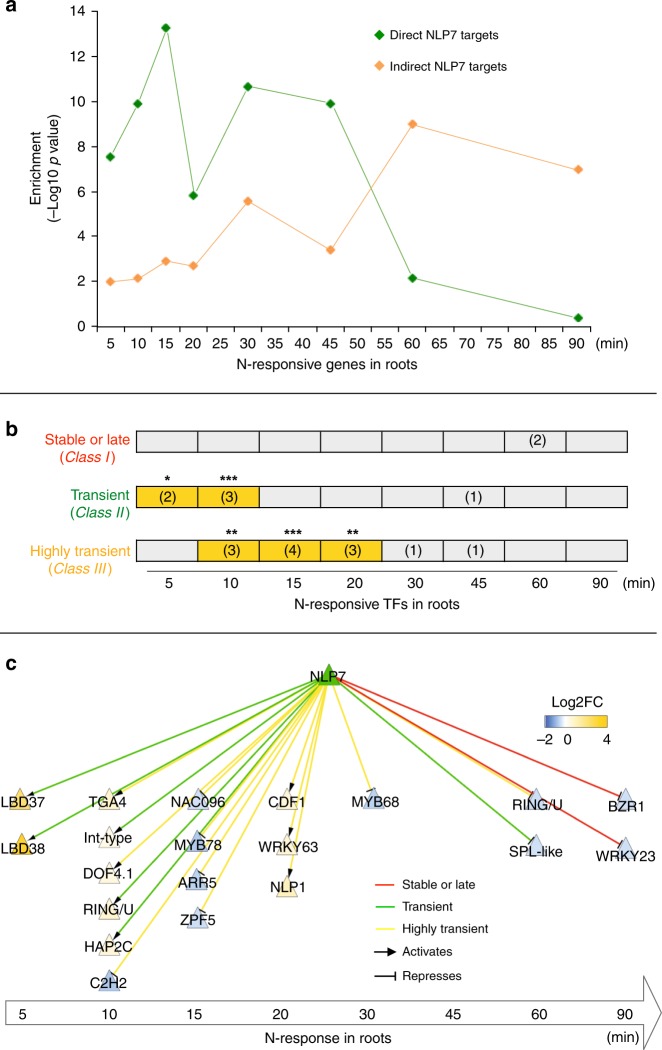
NLP7-direct targets are enriched in early N-responsive genes including secondary TFs. **a** Intersection of NLP7-direct and indirect targets detected in root cells with a time-series of N-response genes in whole roots^19^. The time points represent the just-in-time analysis^19^ which binned genes based on the first time point at which their mRNA levels were affected by N treatment at 5, 10, 15, 20, 30, 45, 60, and 90 min. The significance (*p*-value) of the intersection between NLP7 targets with each N-time point was calculated and −log10 (*p*-value) was graphed. **b** Intersection of N-responsive secondary TFs regulated by NLP7 and genes belonging to each class of NLP7 binding. Size of overlap is listed in parentheses, and significance is indicated by yellow highlighting and asterisks (Fisher’s exact test, *p-value< 0.05; **p-value < 0.01; ***p < 0.001). **c** The transcriptional network regulated by NLP7. NLP7 directly regulates the expression of N-responsive secondary TFs enriched in early time points of the N-response in roots. Node color depicts changes of transcript abundance of TFs in response to NLP7 nuclear import (+DEX/−DEX) (Supplementary Data 5). Edge color corresponds to the different NLP7 mode of actions.

We next examined whether and how the transient targets of NLP7 might play a role in the dynamics of the N-response cascade. We thus quantified the extent to which the transient targets of NLP7 were represented in a time-series transcriptome from a study which binned genes based on the first time point their mRNA levels were affected by an N treatment in whole roots^19^, comparable with the N treatment used in our present study. The NLP7-direct regulated targets identified in root cells using TARGET were significantly and specifically enriched for early N-responsive genes (e.g., 5–20 min), compared with late N-responsive genes (e.g., 60 and 90 min) identified in whole roots (Fig. 3a). Moreover, NLP7-regulated secondary TFs are also enriched in these early N-response genes (Fig. 3b). Among these NLP7-regulated secondary TFs (62 TFs) (Supplementary Data 6), 32% were N-responsive (20/62). Of the N-responsive secondary TFs, 75% (15/20) are categorized as early N-responsive (e.g., 5–20 min), and 25% (5/20) as late N-responsive (Fig. 3b, c). Impressively, 75% of early N-responsive NLP7-regulated TFs (15/20) were transient or highly transient NLP7 targets (Fig. 3b, c).

NLP7’s transient interactions with, and regulation of a significant number of early N-response secondary TFs (Figs. 3b, c), suggested a signaling cascade triggered by NLP7 action via these early secondary TF-transient targets. To test this hypothesis, we validated NLP7-direct vs. indirect targets by comparing NLP7 function in root cells using TARGET—in the presence or absence of CHX. Direct NLP7 targets (492 genes) were identified as NLP7-regulated in both +CHX and −CHX datasets (Supplementary Data 5), while indirect NLP7 targets (2059 genes) were regulated only in −CHX samples (Supplementary Data 18). We found that the direct targets of NLP7 identified in root cells were specifically enriched in early N-response genes in whole roots^19^ (Fig. 3a). By contrast, indirect NLP7 targets identified in root cells were specifically enriched only in the late N-response gene sets (e.g., 60 and 90 min) in whole roots^19^ (Fig. 3a). Collectively, our time-based analyses suggest that NLP7 transient binding and activation of early N-responsive secondary TF targets initiates the early events in a temporal transcriptional cascade that amplifies the transcriptional output of NLP7 during the N-response.

### Transient TF targets of NLP7 mediate downstream responses

**Fig. 4:**
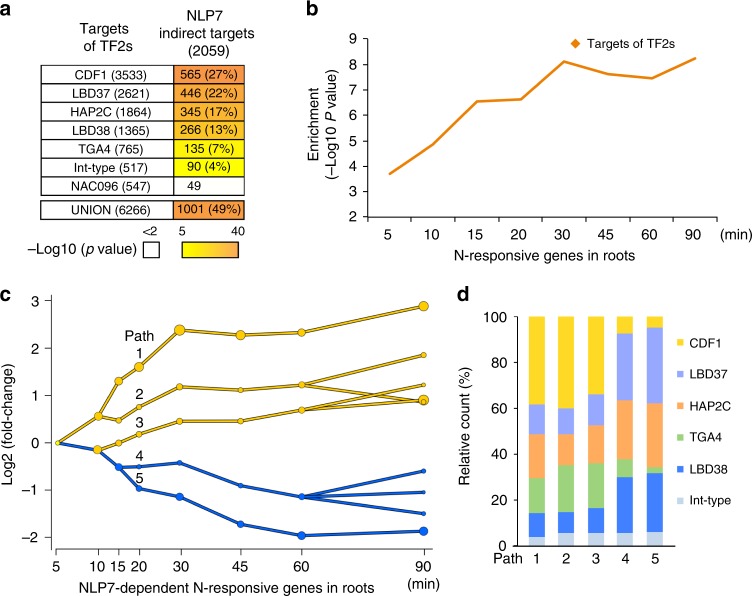
NLP7-regulated secondary TFs mediate downstream effects of NLP7 in the N transcriptional cascade. **a** Intersection of direct targets of validated secondary TFs with NLP7 indirect targets (2059 genes). The significance (*p*-value) of each intersection was calculated and −log10 (*p*-value) was used for the heatmap. **b** Intersection of the union of direct targets of secondary TFs identified in root cells, with N-response genes in whole roots^19^. The significance (*p*-value) of the intersection between direct targets of secondary TFs with each time point was calculated and −log10 (*p*-value) was graphed. **c** DREM2^34^ reconstructed RNA expression NLP7-dependent paths 90 min post N treatment. Each path corresponds to a set of genes that are co-expressed. Split nodes (yellow and blue circles) represent a temporal event where a group of genes co-expressed up to that point diverge in expression, most likely due to regulatory events. **d** The contribution of secondary TFs mediating each DREM2 path were assessed by intersecting genes from each path with genes induced or repressed by each secondary TF.

The transient targets of NLP7 were specifically enriched in N-early response TFs in the temporal N-response cascade (Fig. 3b, c). We thus set out to test the hypothesis that these early and transient secondary TF targets of NLP7 themselves directly mediate the transcriptional responses downstream of NLP7. To this end, we experimentally validated the direct genome-wide targets of seven of these transient early N-response secondary TF targets of NLP7 using the TARGET system in root cells from *nlp7* mutants (Fig. 4a). These early N-responsive secondary TFs included four TFs that have been previously validated in the N-response in planta (e.g., LBD37 & LDB38^18^, CDF1^19,21^, TGA4^20,21^), and three TFs with no prior known role in N-signaling—HAP2C, NAC096, and Integrase-type DNA (At4g39780). Following transfection of the GR-TF plasmids, root cells were sequentially treated with N (20 mM KNO_3_ + 20 mM NH_4_NO_3_) for 100 min and 35 µM CHX for 20 min before a 10 µM DEX treatment to induce TF nuclear entry. The number of direct genome-wide targets for each of the seven secondary TF-transient targets of NLP7 validated in the TARGET system was between 517 genes (Integrase-type DNA) and 3533 genes (CDF1) (Fig. 4a, first column; Supplementary Data 19–25). The direct targets of 6/7 of these secondary TFs had a broad and significant overlap with NLP7 indirect targets (Fig. 4a, second column). The number of secondary TF direct targets that overlap with NLP7 indirect targets varied for each secondary TF, with CDF1 showing the highest overlap with NLP7 indirect targets (27%) (Fig. 4a, second column). NAC096 was the exception, which was the only secondary TF tested that was repressed by NLP7 (Figs. 3c, 4a). Collectively, these transient secondary TF targets of NLP7 which we validated directly targeted ~ 50% of the NLP7 indirect targets (Fig. 4a, second column, union).

Next, we asked how the direct targets of the secondary TFs that we validated in root cells using TARGET assays compared with in planta studies of the N-response. We found a high and significant overlap of the direct targets of the NLP7-regulated secondary TFs with N-responsive genes in whole roots^19^ (Supplementary Fig. 9). We also found a significant overlap of the secondary TF targets identified in root cells with their targets identified in planta using data from 35S:LBD37 and 35S:LBD38 overexpression lines^18^ (Supplementary Fig. 9). Moreover, the union of direct targets of the secondary TFs, CDF1, LBD37, HAP2C, LBD38, TGA4, and Integrase-type DNA, comprise a significant and large fraction 44% (1194/2681) of N-responsive genes in whole roots^19^ (Supplementary Fig. 9). Indeed, the collective direct targets of these secondary TFs acting downstream of NLP7 showed enrichment in N-responsive genes that increased progressively with time, with lowest enrichment at 5 min and highest enrichment after 30 min (Fig. 4b). These results suggest that these early and transient secondary TF targets of NLP7 regulate later downstream transcriptional responses to N treatment in roots. N-responsive genes controlled by these secondary TFs were significantly enriched in gene ontology (GO) terms related to carbohydrate and phosphorous metabolic process, and amino acid transport, among other N-related processes (Supplementary Data 26).

We next examined the extent to which the NLP7-dependent transcriptional cascade mediates the N-response in roots of whole plants. Collectively, NLP7-direct targets, NLP7 indirect targets and genes regulated by secondary TFs acting downstream of NLP7 regulate 53% of the N-responsive genes in whole roots (1420/2681 genes; *p*-value 1.41E-21, Fisher’s exact test; Supplementary Data 27). These genes include the signaling cascade that links the nitrate-mediated regulation of Ca^2+^-sensor protein kinases (CPK) to transcriptional regulation via NLP7^13^. Specifically, the NLP7 cascade that we validated captured 53% of genes dependent on the CPK pathway (164/310 genes; *p*-value 2.58E-24, Fisher’s exact test)^13^.

Finally, to assess the impact of NLP7 transient targets on the dynamic N-response cascade in whole roots, we projected the dynamics of the NLP7-dependent transcriptional cascade using the Dynamic Regulatory Events Miner 2 (DREM2)^34^. The results of this analysis suggest that major regulatory events—indicated by the bifurcations— occur starting at 5 min and up to 20 min into the N response, consistent with the activation of secondary TFs by NLP7 at early time points in the N-response cascade (Fig. 3c). This DREM time-series analysis revealed that NLP7 mediates five main paths during the dynamic N response. Three of these paths are associated with gene activation (Fig. 4c, paths 1–3) and two paths with gene repression (Fig. 4c, paths 4 and 5) in response to N treatment of whole roots (Fig. 4c). Based on our validation studies in root cells using TARGET, this analysis showed that secondary TFs acting directly downstream of NLP7: CDF1, HAP2C, and TGA4 were the main TFs mediating gene activation in the dynamic N-response in whole roots (Fig. 4d, paths 1, 2, and 3); while HAP2C, LBD37, and LBD38 were the main TFs mediating gene repression by N treatment (Fig. 4d, paths 4 and 5). These results are consistent with previous in planta studies showing that LBD37 and LBD38 act as transcriptional repressors in response to N treatment^18^.

**Fig. 5:**
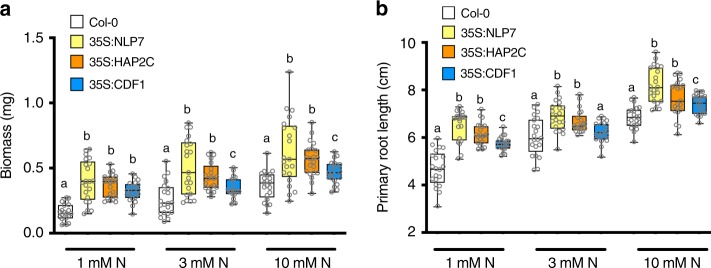
Overexpressing early and transient TF targets of NLP7, HAP2C, and CDF1 significantly increases plant biomass and primary root growth. Col-0, 35S:NLP7, 35S:HAP2C, and 35S:CDF1 plants were grown for 2 weeks on vertical plates containing different concentrations of N. **a** Plant growth of *NLP7*, *HAP2C*, and *CDF1* overexpressor lines is enhanced as compared with wild-type Col-0 plants under increasing N concentrations. **b** Primary root growth of *NLP7*, *HAPC*, and *CDF1* overexpressor lines is enhanced as compared with wild-type plants under increasing N concentrations. Data are from three independent experiments (*n* = 21). Different letters indicate mean values differ significantly between genotypes in each condition (*t* test, *p*-value < 0.05). Source data are provided as a Source Data file.

The NLP7-regulated secondary TFs—LBD37, LBD38, and TGA4—have been previously identified to regulate gene responses to N in planta, as well as N-related growth phenotypes^18,20^. We thus decided to evaluate the functional relevance of NLP7-regulated secondary TFs that our study identified, which had no prior known role in regulating plant growth in response to N. To this end, we selected the NLP7-regulated secondary TFs—CDF1 and HAP2C— for functional validation in planta, based on the high overlap of their direct target genes with NLP7 indirect targets, identified in root cells using the TARGET system for TF perturbation (Fig. 4a). These findings support the notion that CDF1 and HAP2C are major secondary TFs acting downstream of NLP7 in the N-signaling cascade. To test their significance to the N-response in planta, we compared the growth phenotypes of overexpression lines for *NLP7*^13^*, HAP2C*^35^, and *CDF1*^36^ under three different N concentrations, compared with Col-0 plants. Consistent with previous studies^37^, we found that *NLP7* overexpression leads to significant increases in plant biomass (Fig. 5a) and primary root length, at all three N concentrations, compared with Col-0 (Fig. 5b). Importantly, the overexpression of secondary TFs acting downstream of NLP7—35S:HAP2C and 35S:CDF1—also showed significant increases in biomass (Fig. 5a), and primary root growth, as compared with Col-0 plants in these conditions (Fig. 5b). These results support the notion that HAP2C and CDF1 act downstream of NLP7 in mediating the plant growth responses to N.

**Fig. 6:**
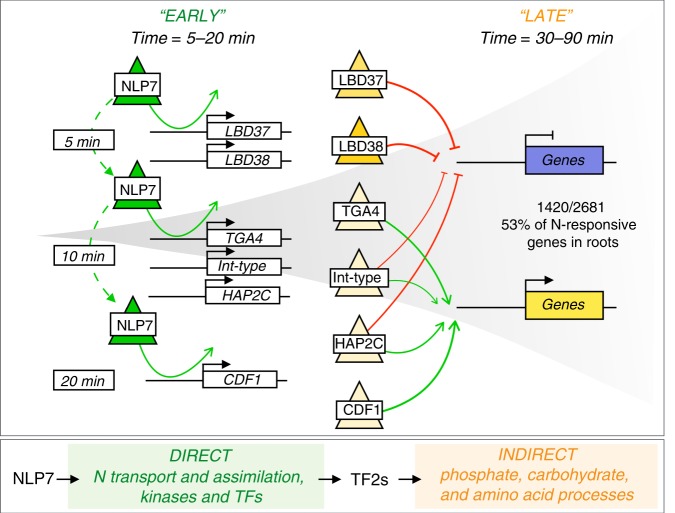
A validated model for NLP7 role in rapidly initiating a cascade that amplifies the downstream N-response. Transient interactions of NLP7 initiate early N-response genes including genes involved in N-uptake and early N-response TFs. The transient interactions of NLP7 enable it to rapidly activate secondary TFs leading to a transcriptional burst in a short period of time. Secondary TFs amplify the NLP7-initiated cascade by regulating downstream late N response genes enriched in phosphate, carbohydrate, and amino acid processes (Supplementary Data 26). LBD37 and LBD38 primarily mediate transcriptional repression, and both have been shown to have in planta relevance^18^; CDF1 and TGA4 primarily mediate transcriptional activation; and Int-type and HAP2C act as either gene activators or repressors depending on the target downstream of NLP7. Collectively, the direct targets of NLP7 and direct targets of secondary TFs account for 53% of the N response in plant roots^19^.

Taken together, the validated targets for NLP7 and its downstream secondary TFs indicate that the NLP7 cascade directly and indirectly modulates a large and significant proportion of the dynamic changes in gene expression triggered by N supply to roots (Fig. 6). Unexpectedly, we found that the transient and highly transient interactions of NLP7 with its genome-wide targets specifically activates early N-response secondary TFs, which in turn amplify and control common and divergent aspects of the dynamic transcriptional response to N supply (Fig. 6). Collectively, the direct targets of NLP7 and direct targets of secondary TFs account for 53% of the N response in plant roots^19^ (Fig. 6).

## Discussion

This study highlights an important but overlooked feature of gene-regulatory networks—the transient mode of action of transcription factor target binding and regulation genome wide. Moreover, we show that these typically overlooked transient TF targets are important in initiating early events in an N-signaling cascade, which amplify later downstream outcomes.

Our findings highlight the importance of transient TF–target interactions which are typically missed. As TF perturbations typically cannot distinguish primary from secondary targets, the current gold standard in the field requires that genes that are TF-bound and TF-regulated to be considered bona fide direct targets of that TF. The TF-regulated but not bound genes are typically dismissed as indirect targets. Our ability to capture direct targets for NLP7 based on gene regulation only, and to confirm transient binding by time-series ChIP and/or DamID, support a more interesting alternative. Namely, that the large proportion of TF-regulated but not detectably bound genes in planta^11^ are in fact transiently bound by NLP7, when assayed in root cells at early times after TF nuclear entry. We also show that these transiently NLP7-bound targets are crucial to the early N-response, as discussed below.

We focused on NLP7, a master TF in the N-response pathway^11,13^, as a paradigm for the two paradoxes of the low overlap of TF-binding and TF-regulation genome-wide datasets. Paradox 1: Despite the large and extensive TF binding to genome-wide targets, across eukaryotes only 5–40% of TF-regulated targets are bound by the TF^1–6,14^. Paradox 2: The vast proportion of TF-bound genes detected in vivo are not TF-regulated^3,8,38^. NLP7 exemplifies both paradoxes—it binds to only ~10% of the NLP7-regulated genes in *Arabidopsis* roots, and the vast portion of NLP7-bound genes detected in planta are not NLP7-regulated^11^. We solved Paradox 1, by exploiting a cell-based TF-perturbation system that can identify direct TF targets either by TF regulation or TF binding (stable or transient). We found that a large and significant proportion of NLP7-regulated but not detectably bound genes in planta could be explained by NLP7 transient or highly transient interactions detected in root cells. Indeed the transient TF–target interactions that we captured in root cells using time-series ChIP or DamID, account for 50% of the NLP7-regulated in planta responses where TF binding was missed^11^. Thus, the transient mode of action for NLP7 uncovered at early times following controlled nuclear entry of TF using the plant cell-based TARGET TF-perturbation system helped refine our understanding of the in planta mechanism of NLP7. Our findings indicate that the transient mode of action of a TF is an underestimated mechanism that reflects an important—but overlooked—component of dynamic GRNs in whole organisms.

We were able to capture these elusive transient TF–target interactions in root cells using a combination of two complementary approaches; time-series ChIP and/or DamID (Fig. 2; Supplementary Fig. 5). Technical differences may explain why the two methods are complementary and not completely overlapping. ChIP relies on biochemically cross-linking DNA and proteins, followed by shearing of DNA into fragments, typically by sonication. In the DamID technique, adenine methylation of target genes touched by the Dam-TF construct occurs in vivo, and DNA can be extracted from unfixed or even live cells^39^. However, the resolution of DamID depends on the distribution of GA^me^TC sites in the genome, which can be identified by DpnI digestion. DamID also differs from ChIP, as the latter relies on the specificity and high affinity of antibodies. More importantly, the temporal nature of TF binding emphasizes a fundamental difference between the two protocols, namely that ChIP measures when and where a TF is bound to DNA at any one time point analyzed, whereas DamID leaves a lasting methylation mark on the DNA which indicates whether a TF has been in proximity to the DNA sequence, at any time point, even if only transiently (Fig. 2)^17,40^. Thus, the approaches are complementary.

It is also noteworthy that the number of NLP7-bound genes identified by either time-series ChIP (Supplementary Data 1–4) or DamID (Supplementary Data 11), vastly exceeded the number of genes directly regulated by NLP7 in root cells. While this may seem to suggest that the specificity of TF-target binding approaches such as ChIP or DamID is low, the actual specificity is difficult to estimate. This is because TF-bound genes that are not transcriptionally activated may be poised, and awaiting cofactors or other conditions to induce a change in expression. This is an easy explanation for Paradox 2, as has been described extensively for ChIP in planta and across eukaryotes^3,8^.

What is the long-term effect of transient TF binding? We showed that transient NLP7-binding lead to sustained transcription. Using 4tU labeling of nascent transcripts, we showed that transient interactions of NLP7 with its targets initiated de novo transcripts affinity captured at time points when NLP7 is no longer bound (e.g., 3 h) (Figs. 1, 2). Collectively, these findings validate a central principle of a Hit-and-Run transcription model initially proposed in 1988^41^ and validated for bZIP1^14,16^—and now for NLP7, a second master TF on a genome-wide scale. Importantly, for NLP7 our DamID studies now conclusively prove that the TF was transiently bound to a set of actively transcribed targets.

Our studies on the dynamic role of NLP7-target binding also provide insights into the timing and early steps of the N-signaling pathway. Once nitrate enters the cell through the NRT1.1 transporter, Ca^2+^ waves are induced through the action of a yet to be identified phospholipase C^42^. This Ca^2+^ signal is decoded through CPKs from the subgroup III, which phosphorylate NLP7 and force it to remain in the nucleus where it activates N-responsive genes^13^. The timing of this Ca^2+^ wave takes seconds to respond to N supply and subsequent NLP7 nuclear retention, controlled by NLP7 phosphorylation, follows shortly after, allowing the TF to regulate gene expression within minutes^11,13^. As a result of this cascade, NLP7 rapidly controls the expression of TF genes, suggesting that NLP7 can induce a broad outcome through transcriptional cascades^11,12^. The advantage of a posttranscriptional activation mechanism coupled with a Hit‐and‐Run activation mechanism allows an organism to rapidly alter its genetic program with a small number of TF molecules and without the delay of a multistep transcriptional cascade.

Our results support a scenario in which rapid and highly transient NLP7-target interactions directly activate downstream regulatory circuits controlled by secondary TFs, which in turn amplify common and specific aspects of the transcriptional output in response to N. This suggests that transient interactions of a master TF can produce a large transcriptional burst of gene expression in a remarkably short period (Fig. 6). Thus, small changes in expression of a subset of genes directly controlled by NLP7’s activity have an expansive effect on N responses genome wide. This highlights the upper hierarchical role of NLP7 as a main coordinator of N-signaling, as suggested previously on the basis of stable ChIP detectable binding^11^, but indeed largely underestimated when taking into account the transient nature of the TF–DNA interactions. Beyond their implications for nitrogen signaling and nitrogen use efficiency, our approaches to capture and model transient TF–target interactions on a genome-wide scale are broadly applicable to capture dynamic interactions in GRNs relevant to biology, agriculture, and medicine.

## Methods

### Plant materials for the NLP7 TARGET assay

*Arabidopsis thaliana nlp7* mutant seeds^11,23^ were the source for the root cells used for the TARGET assay of NLP7 activity. Seeds were vapor-phase sterilized, vernalized for 3 days, then 1 mL of seed was sown on 12 agar plates containing 1% w/v sucrose, 0.5 g/L MES, 1× MS basal salts (-CN, Phytotechnology Laboratories, http://www.phytotechlab.com), 1 mM KNO_3_, 2% agar, pH 5.7 for 10 days prior to the TARGET experiment. Plants were grown vertically on plates in a plant growth incubator (Percival Scientific, Perry, IA), whose light regime was set to 120 µmol m^−2^ s^−1^ and 16 h light/8 h dark at constant temperature of 22 °C.

### TARGET NLP7-perturbation assays

The pBOB11_C-Term vector (NCBI GenBank nucleotide database under accession MN991175) is a derivative of pBOB11^22^ and pBeaconRFP^43^. This empty vector can be used create a TF–GR C-terminal fusion for the TARGET TF-perturbation assay^22^. To this end, the NLP7 coding sequence was amplified and cloned into pENTR and then subcloned into the destination empty vector pBOB11_C-Term by LR recombination (Life Technologies). This NLP7-GR vector construct was used in the cell-based TARGET assay as described in Bargmann et al.^22^. Briefly, *Arabidopsis nlp7* seedlings were grown on vertical plates in MS basal salts (-CN) media supplemented with 1 mM KNO_3_ and 1% w/v sucrose under 120 μmol m^−2^ s^−1^ light condition of 16 h light, 8 h dark, and constant temperature at 22 °C. After 10 days, 2 h after subjective dawn, plant roots were harvested, finely cut, and placed in protoplast solution for 3 h. Root cell protoplasts were washed, and then polyethylene glycol 4000 (PEG) mediated transfected with a pBOB11_C-Term vector containing NLP7. For each assay, 4–6 million cells were transfected. Transfected root cells were incubated overnight. Protoplast suspensions were treated sequentially with: N supply as in MS media (20 mM KNO_3_ + 20 mM NH_4_NO_3_) for 100 min, then 2) either +CHX (35 µM in DMSO, Sigma-Aldrich) or −CHX (DMSO alone) as mock for 20 min, and then 3) with either +DEX (10 µM in EtOH, Sigma-Aldrich) or −DEX (EtOH alone) as mock for 180 min at room temperature. Treated root cell protoplast suspensions were sorted using fluorescence-assisted cell sorting (FACS) as in Bargmann et al.^22^. Approximately 10,000 RFP-positive cells were FACS sorted directly into RLT buffer (QIAGEN) for RNA extraction. Cells overexpressing NLP7 were collected in triplicate, and RNA-Seq libraries were prepared from their mRNA using the NEBNext® UltraTM RNA Library Prep Kit for Illumina®. The libraries were pooled and sequenced on the Illumina NextSeq 500 platform for 75 cycles.

### Identification of NLP7-direct or indirect targets

The RNA-Seq reads from the NLP7 TARGET experiments were aligned to the Araport11 genome assembly using TopHat2^44^ and gene expression estimated by the GenomicFeatures/GenomicAlignments packages^45^. To identify NLP7-direct targets, the gene counts for every sample were combined, and differential expression by NLP7 nuclear import was calculated between +DEX libraries and −DEX libraries in the samples pretreated with +CHX by using the DESeq2^46^ package. The raw *p*-value of differentially expressed genes was adjusted by false discovery rate (FDR) to control for multiple testing. Genes significantly induced or repressed by NLP7 were then selected with an FDR cutoff of 10%. To eliminate any gene expression artifacts caused by +CHX pretreatment, we only considered genes in our NLP7-direct target analysis that responded to DEX-induced NLP7 nuclear import in both the presence or absence of CHX. Using this filter, we identified 492 direct regulated NLP7 targets (Supplementary Data 5). To identify NLP7 indirectly regulated targets, differential expression by NLP7 nuclear import was calculated between +DEX and −DEX libraries in the samples without CHX pretreatment (solvent alone) using the DESeq2^46^ package. The raw *p*-value of differentially expressed genes was adjusted by FDR to control for multiple testing. Genes significantly induced or repressed by NLP7 were then selected with an FDR cutoff of 10%. This list contains genes that are direct or indirect targets of NLP7. By discarding genes identified as direct NLP7 targets (Supplementary Data 5), we obtained 2059 genes as indirect NLP7-regulated targets (Supplementary Data 16).

### Affinity capture of de novo mRNAs in 4tU-labeled fractions

Here we used 4tU to identify the de novo transcripts initiated by NLP7 in the TARGET system, as described in Doidy et al.^16^. Root protoplasts were prepared, transfected, and sorted as described above. Cells transfected with NLP7 vector were treated sequentially with: N supply as in MS media (20 mM KNO_3_ + 20 mM NH_4_NO_3_) for 100 min, then CHX (35 µM in DMSO, Sigma-Aldrich) and then with either +DEX (10 µM in EtOH, Sigma-Aldrich) or −DEX (solvent alone) as mock for 180 min at room temperature. 4tU labeling and purification of 4tU-labeled fractions were done as described in Doidy et al.^16^. RNA-seq libraries were prepared from triplicates of +DEX and −DEX samples using the NEBNext® UltraTM RNA Library Prep Kit for Illumina®. The libraries were pooled and sequenced on the Illumina NextSeq 500 platform for 75 cycles. The RNA-seq reads were aligned to the Araport11 genome assembly using TopHat2^44^, and gene expression was estimated using the GenomicFeatures/GenomicAlignments packages^45^. To identify actively transcribed (4tU-labeled fractions) NLP7-direct targets, differential expression by NLP7 nuclear import was calculated between +DEX libraries and −DEX libraries using the DESeq2^46^ package. The raw *p*-value of differentially expressed genes was adjusted by FDR to control for multiple testing. Genes significantly induced or repressed by NLP7 were then selected with an FDR cutoff of 10% (Supplementary Data 15).

### Chromatin immunoprecipitation sequencing

Protoplast suspensions containing ~10,000 NLP7-GR transfected *nlp7* root cells were treated sequentially with: N supply as in MS media (20 mM KNO_3_ + 20 mM NH_4_NO_3_) for 100 min, then CHX (35 µM in DMSO, Sigma-Aldrich) and then with +DEX (10 µM in EtOH, Sigma-Aldrich) for 5, 10, 30, or 180 min at room temperature. For this analysis, we included a control at time 0 before +DEX treatment. Root cells were incubated with gentle rotation in 1% formaldehyde in W5 buffer for 7 min, then washed with W5 buffer, and frozen in liquid N_2_. A detailed protocol of Micro-ChIP is described in Para et al.^15^. Briefly, the NLP7-GR-DNA complexes were captured using 2.5 µg of anti-GR antibody (GR P-20, Santa Cruz biotech) bound to Protein-A beads (Life Biotechnologies). The complexes were washed twice with RIPA buffer (10 mM Tris-HCl, pH 7.5 m 140 mM NaCl, 1 mM EDTA, 0.5 mM EDTA, 1% (vol/vol) Triton X-100 and 0.1% (wt/vol) SDS); once with LiCl wash buffer (0.25 M LiCl, 1% (wt/vol) Na deoxycholate, 10 mM Tris-HCl pH 8, 1% NP40, 1 mM EDTA); and once with TE buffer (10 mM Tris-HCl and 10 mM EDTA). After elution from the beads, the ChIP material and the Input DNA for each time point were cleaned and concentrated using QIAGEN MiniElute Kit (QIAGEN). Libraries from the ChIP DNA and Input DNA for each time point were prepared using the NEBNext® Ultra II DNA Library Prep Kit for Illumina®. The quality and concentration of the libraries were determined using the Agilent 2200 TapeStation System and the KAPA Quant Library Kit for Illumina (KAPA Biosystems, MA, USA), respectively. A total of 12 libraries were then pooled in equimolar amounts and sequenced on the Illumina NextSeq 500 platform for 75 cycles.

### ChIP-seq analysis of NLP7 targets

Reads obtained from the ChIP DNA and Input DNA for each time point were filtered and aligned to the Araport11 genome using Bowtie2^47^ and clonal reads were removed. The ChIP alignment data was compared with its partner Input DNA and peaks were called using MACS2 (*q* = 0.05)^48^. These peaks were overlapped with the genome annotation to identify genes within 2 kb downstream of the peak using BEDTools^49^. In all, 6288, 1299, 1518, and 861 were identified as NLP7-bound by ChIP at 5, 10, 30, and 180 min, respectively (Supplementary Data 1–4). For each NLP7-binding region mapping to genes, the reads per kb of binding site per million sample reads (RPKM) were calculated using deepTools 2.0^50^ (Supplementary Fig. 2). The filtered, sorted, and scaled bam files were converted to the bigwig format using the “bamCoverage” script in deepTools 2.0^50^ with a bin size of 10 bp and RPKM normalization. Heatmaps and average plots displaying ChIP-seq data across time points (Supplementary Fig. 2) were generated using the “computeMatrix”, “plotHeatmap”, and “plotProfile” functions in the deepTools 2.0 package. Genome browser images were made using the Integrative Genomics Viewer (IGV)^51^ (Figs. 1b,  2d).

### DNA adenine methylation IDentification

We engineered a pDamBOB expression plasmid that contains no DpnI sites (GATC) (NCBI GenBank nucleotide database under accession MN956899) based on the pBOB11^22^ and pBeaconRFP^43^ vectors. We did this to address the problem that the TF-expression vector containing GATC sites is methylated and cut by DpnI. This bacterial DNA contaminates the resulting Illumina libraries, and reduces the quantity of plant genomic DNA captured. Thus, the GATC-depleted pDamBOB expression vector was generated as follows. An intermediate vector containing only six GATC sites within the origin of replication was made by synthesizing the construct in three fragments and fusing them together using traditional restriction enzyme cloning. The ColE1 origin of replication in this intermediate vector was then replaced with the R6K gamma ori, a low copy, stringent origin of replication that contains no GATC sites. To clone NLP7 into this vector and in frame with the Dam-GR, the coding sequence of NLP7 with a stop codon was synthesized to remove all GATC sites (Supplementary Data 28) and add BsaI restriction sites to either end. The overhangs produced by BsaI digest of the NLP7 fragment were compatible with those made by digesting pDamBOB with BsaI.

Root protoplasts from *nlp7* mutants were prepared and transfected with the pDamBOB vector containing NLP7, and treated as described above as for the ChIP experiment. +DEX treatment (in 10 µM in EtOH, Sigma-Aldrich) was done for 180 min at room temperature. For this analysis, we included a pDamBOB empty vector control with Dam but no TF (Dam-only) to subtract nonspecific background DNA adenine methylation, as reported in Gutierrez-Triana et al.^32^. Genomic DNA was extracted from cells using the DNeasy Plant Mini kit (Qiagen). The generation of DamID libraries was done as reported in Gutierrez-Triana et al.^32^. Briefly, in a 20 µL reaction, 2 µL 10× NEB3.1 buffer, 50 ng of gDNA and 10 units of *Dpn*II (NEB, R0543S) were mixed and incubated for 6 h at 37 °C. The enzyme was inactivated by incubation at 65 °C for 20 min. To the inactive *Dpn*II reaction, 3 µL H_2_O, 1.5 µL 10× NEB CutSmart buffer and 0.5 µL of Quick CIP (NEB, M0510S) were added, then the mixture was incubated for 1 h at 37 °C. The reaction was cleaned up using the Genomic DNA Clean and Concentrator −10 (Zymo Research) and eluted in 10 µL of 10 mM Tris-HCl pH 7.5. In a 15 µL reaction, 1.5 µL NEB CutSmart buffer, 10 µL of *Dpn*II/AP-treated sample and 10 units of *Dpn*I enzyme (NEB, R0176S) were mixed. The reaction was incubated at 37 °C for 6 h, then the reaction was cleaned up using Genomic DNA Clean and Concentrator −10 (Zymo Research) and eluted in 10 µL of 10 mM Tris-HCl pH 7.5. In a 20 µL reaction, 10 µL of treated samples, 4 µL 5× Quick Ligation Reaction Buffer ((NEB, 6058 S), 1 µL of 50 µM dsOligos AdRt/AdRb^32^, 0.5 µL of T4 DNA ligase (NEB, M0202L), and 4.5 µL H_2_O were mixed. The reaction was incubated overnight at 16 °C and cleaned up using Genomic DNA Clean and Concentrator −10 (Zymo Research) and eluted in 50 µL of 10 mM Tris-HCl pH 7.5. 25 µL of ligation sample, 5 µL of 10× Advantage 2 PCR buffer (Takara, S1799), 1 µL of 50× Advantage 2 Polymerase Mix (Takara, S1798), 1 µL of dNTPs mix (Takara, 4030), 1 µL 10 µM AdR_PCR primer^32^ were added to a 50 µL reaction. PCR was carried out as follows: 68 °C for 10 min; 1 cycle of 94 °C for 15 s, 65 °C for 30 s, and 68 °C for 5 min; and 20 cycles of 94 °C for 15 s, 65 °C for 30 s, and 68 °C for 2 min. Each PCR sample (5 µL) was run on a 1% agarose gel to confirm the presence of a smear in the DamID samples (around 200 bp to 2 kb). As the optimal range of DNA fragments for Illumina sequencing is ~200–500 bp, DNA samples were fragmented using the Covaris S2 sonicator in AFA fiber microtubes (Covaris, 520045). Libraries from the NLP7-DamID DNA and Dam-only DNA were prepared using the NEBNext® Ultra II DNA Library Prep Kit for Illumina®. The quality and concentration of the libraries were determined using the Agilent 2200 TapeStation System and the KAPA Quant Library Kit for Illumina (KAPA Biosystems, MA, USA), respectively. Libraries were then pooled in equimolar amounts and sequenced on the Illumina NextSeq 500 platform for 75 cycles.

### DamID-seq analysis of NLP7-direct targets

Reads obtained from the NLP7-DamID and Dam-only samples were filtered and aligned to the Araport11 genome using Bowtie2^47^ and clonal reads were removed. The NLP7-DamID alignment data was compared with the Dam-only DNA, and peaks were called using MACS2 (*q* = 0.05)^48^. These peaks were overlapped with the genome annotation to identify genes within 2 kb downstream of the peak using BEDTools^49^. This analysis led to the identification of 8625 genes (Supplementary Data 11). In Fig. 2c, for NLP7-bound genes by DamID, the log2 ratio NLP7-DamID/Dam-only of the bam files was calculated using the “bamCompare” script in deepTools 2.0^50^ with a bin size of 10 bp and with a scaling factor that accounted for the different number of reads in each bam file. The DamID signal plot was generated using the “computeMatrix”, and “plotProfile” functions in the deepTools package^50^. Genome browser images were made using the Integrative Genomics Viewer (IGV)^51^ (Fig. 2d).

### GO enrichment analysis

Fisher’s exact test was performed for declaring a GO (Gene Ontology) category as significantly overrepresented (Benferroni method for controlling FDR, adjusted *p*-value < 0.01)) using the PlantGSEA toolkit^52^. In order to focus on specific functions, we removed redundant terms using the REVIGO tool and the Lin option as a semantic similarity measure^53^.

### Cis-binding site enrichment

To search for the enrichment of the NLP7 cis-motif in promoters of genes from each NLP7 class (Supplementary Fig. 8) or NLP7-bound genes by DamID or ChIP, first the consensus position weight matrix (PWM) for NLP7 was obtained from O’Malley et al.^33^, and was converted to the MEME format. The FIMO tool within the MEME package (http://meme-suite.org/tools/fimo) was used to identify every occurrence of the cis-motif in the 2 kb promoter regions of all *Arabidopsis* genes at a *p*-value < 0.0001. Enrichment of the NLP7 cis-motif in the promoter of genes from each NLP7 class (Supplementary Fig. 8) or bound by NLP7 (ChIP or DamID; Supplementary Data 15 and Supplementary Data 16, respectively) relative to their occurrence in all annotated genes was calculated using a Fisher’s exact test.

To determine the enrichment of potential partner TFs, 2 kb regions upstream of the TSS for NLP7-target genes from the different classes were extracted based on TAIR10 annotation and submitted to the Elefinder program (all promoters from the genome as background) to determine over-representation of known cis-element binding sites. Motifs showing an *E*-value < 0.001 are shown in the heatmap (Supplementary Fig. 8).

### Identification of direct targets of NLP7 secondary TFs

We determined the direct targets of seven secondary TFs directly regulated by NLP7 using the TARGET system^22^ as follows. The seven secondary TFs were TOPO cloned into pENTR (Invitrogen) from cDNA or isolated from the *Arabidopsis* TF collection^54^. TFs were then transferred to the pBOB11 plasmid^22^ or a GFP version of the same plasmid (pBOB11-GFP)^21^ by Gateway (Invitrogen) cloning. Root protoplasts from *Arabidopsis nlp7* mutant plants were prepared, transfected, and sorted as described in Bargmann et al.^22^. For each TF and the empty vector (EV) construct, 3 million cells were transfected separately, and after washing, a single TF in the RFP vector and a single TF in a GFP vector were combined in three replicate wells of a 24-well plate. After overnight incubation, each pool of transfected root protoplasts was treated sequentially with N (20 mM KNO_3_ + 20 mM NH_4_NO_3_) for 100 min and 35 µM CHX for 20 min before a 10 µM DEX treatment to induce TF nuclear entry. Transfected cells were sorted by FACS into GFP and RFP expressing cell populations 3 h after DEX-induced TF nuclear import. Cells overexpressing the TF or EV were collected in triplicate, and RNA-seq libraries were prepared from their mRNA using the NEBNext® Ultra™ RNA Library Prep Kit for Illumina®. The RNA libraries were pooled, sequenced, and mapped as described above. The gene counts for every sample were combined, and differential expression between the TF overexpression libraries and the EV libraries were identified using the DESeq2 package^46^. The raw *p*-value of differentially expressed genes was adjusted by FDR to control for multiple testing. Genes significantly induced or repressed by each TF were then selected with an FDR cutoff of 10% (Supplementary Data 17–23).

### Intersection between gene lists and enrichment analyses

To estimate the significance of the overlap between different gene lists, we first calculated the number of genes in each overlap using the GeneSect analysis tools available at VirtualPlant (www.virtualplant.org)^55^. Next, a Fisher’s exact test was used to determine whether the proportions of genes in the overlap were significantly enriched for each comparison. For example, in Fig. 3a, we calculated the number of NLP7-direct or indirect targets that overlap with genes regulated at different time point according to the time-series analysis of the N-response in roots described by Varala et al.^19^. For this, we calculated the significance of the enrichment for each overlap as compared to the total number of genes regulated at each time point. As a background, we used all genes in the *Arabidopsis* genome that are not N-responsive in roots determined by Varala et al.^19^. We performed this test for every comparison, which means that the groups of genes changed between each test but the background was estimated accordingly.

### DREM analysis

The Dynamic Regulatory Events Miner 2 (DREM2)^34^ uses time-series gene expression data to identify patterns of temporal gene expression. Splits in the reconstructed network (yellow and blue nodes in Fig. 4c) represent divergence of genes that are coregulated up to that point, and can be associated with TF-regulatory events^3,34,38^. We used the DAP-seq TF–target interaction matrix^33^ as prior to run the DREM modeling. The analysis performed here used 1420 N-responsive genes (NLP7-dependent transcriptional cascade (Supplementary Data 25)). We extracted the log2 fold change from the root N time-series RNA-seq data^19^. For Fig. 4d, genes from paths 1, 2, and 3 (associated with gene induction) were intersected with genes induced by each secondary TF; while genes from paths 4 and 5 (associated with gene repression) were intersected with genes repressed by each secondary TF. The numbers on each intersection were used to calculate the relative count percentage for each secondary TF in each path.

### Functional characterization of NLP7 secondary TFs in planta

Seeds from Col-0, 35S:NLP7^13^, 35S:HAP2C^35^, and 35S:CDF1^36^ lines were sterilized with 15% bleach for 12 min, and then washed five times with sterile water. Sterilized seeds were stratified at 4 °C for 2 days, and plated on solid medium containing 1% (w/v) sucrose and 0.8% (w/v) agar. We used MS‐modified basal salt media without N (Phytotechnology Laboratories, http://www.phytotechlab.com), supplemented with KNO_3_ as the sole N source. To maintain the same osmolarity between different N conditions, we prepared the plates as follows: For 10 mM N medium, we used 10 mM KNO_3_; for 3 mM mM N medium, we used 7 mM KCl and 3 mM KNO_3_; and for 1 mM N medium, we used 9 mM KCl and 1 mM KNO_3_. Seeds were germinated, and plants grew at 22 °C under 16-h light/8-h dark photoperiod. Twenty plants per biological replicate were analyzed, and plant images were acquired using an Epson Perfection V700 photo scanner, and primary roots were measured using ImageJ.

### Reporting summary

Further information on research design is available in the Nature Research Reporting Summary linked to this article.

## Supplementary information

Supplementary Information

Reporting Summary

Description of Additional Supplementary Files

Supplementary Data 1-28

## Data Availability

Data supporting the findings of this work are available within the paper and its Supplementary Information files. A reporting summary for this Article is available as a Supplementary Information file. The datasets generated and analyzed during the current study are available from the corresponding author upon request. The sequence of the pDamBOB vector that we used for the DamID-seq experiment is deposited in the NCBI GenBank nucleotide database under accession MN956899. The sequence of the pBOB11_C-Term vector that we used for the TARGET experiment for NLP7 is deposited in the NCBI GenBank nucleotide database under accession MN991175. All pBOB vectors in this study are available by name in the Gateway collection at VIB Gent (https://gatewayvectors.vib.be/). All raw sequencing data from this project is available at the National Center for Biotechnology Information Sequence Read Archive (SRA), with accession number PRJNA555731. The source data underlying Fig. 5 are provided as a Source Data file.

## Source Data

Source Data

## Code availability

The scripts used in this study are available in GitHub.
