# Supplementary material for: Transient genome-wide interactions of the master transcription factor NLP7 initiate a rapid nitrogen-response cascade
Source: Nat Commun. 2020 Mar 2;11:1157. doi: 10.1038/s41467-020-14979-6 (PMC7052136; doi:10.1038/s41467-020-14979-6)
Supplement: Supplementary file 1 — Supplementary Information [file 41467_2020_14979_MOESM1_ESM.pdf]

Transient genome-wide interactions of the master transcription factor NLP7 initiate a rapid nitrogen-response cascade

Alvarez *et al.*

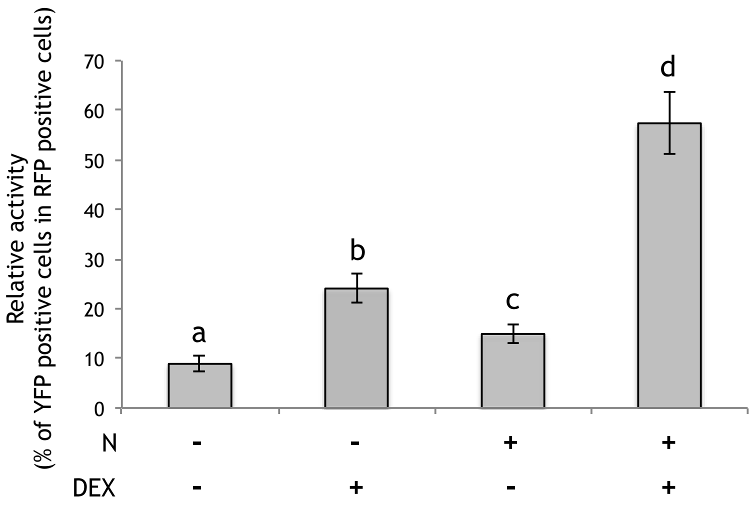

Supplementary Figure 1. N treatment and DEX induced nuclear import of NLP7 act synergistically to activate a Nitrate Responsive Promoter in root protoplasts.

The NRP-YFP reporter construct and 35S-NLP7-RFP effector construct were co-transfected into root protoplast. Protoplast suspensions were treated with 20 mM KNO3 and 20 mM NH4NO3 (N) and/or or DEX (10 µM) for 2 hours. Treated protoplast suspensions were cell-sorted and RFP-positive and YFP-positive cells were counted using the BD Accuri cell counting machine. Activation of NRP reporter by NLP7 in different treatments was estimated by counting the YFP positive cells from the total number of RFP positive cells is each experiment. Values are the means ± standard deviations (n=3). Different letters indicate statistically different means between treatments (p-value < 0.01 by Student's t-test).

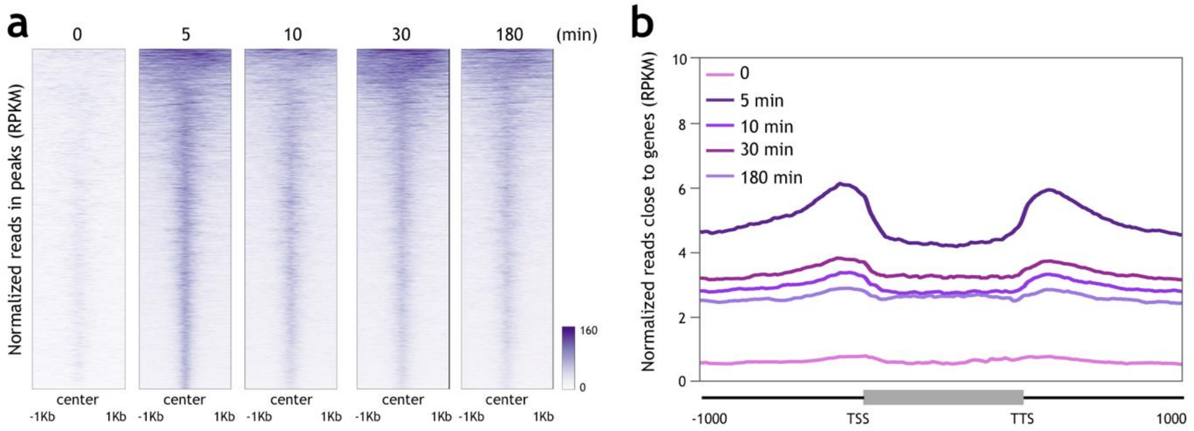

Supplementary Figure 2. NLP7 binding levels as determined by time series ChIP seq.
(**a**) Binding levels of NLP7 are indicated by reads per kilobase per million reads in sample (RPKM) for each genomic location bound by NLP7. RPKM was calculated for NLP7 peaks detected at any time points. Peaks bound by NLP7 were scaled and centered for the heatmap display. (**b**) Binding levels of NLP7 were higher at 5 minutes after nuclear import and NLP7 binding was enriched close to the TSS and TTS of transcribed regions.

a

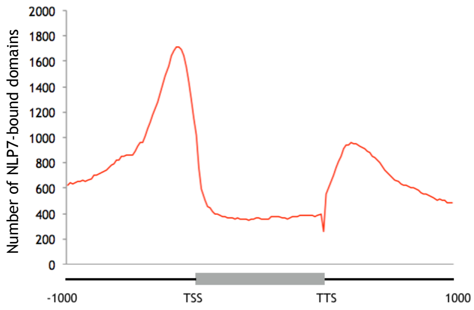

b

	Time series ChIP root cells (6423 genes)			
	# of genes	# of genes in overlap	% of genes in overlap	p-value
NLP7-bound *in planta* (Marchive et al, 2013)	851	483	57%	1.6E-56
N-responsive in roots (Varala et al., 2013)	2681	890	33%	7.3E-4
Nitrate-regulated (Wang et al., 2004)	588	257	44%	1.9E-11

Supplementary Figure 3. NLP7 binding in root cells captured by ChIP have in planta relevance.

(**a**) Spatial distribution and number of NLP7 peaks in proximity to transcribed genes. (**b**) ChIP-seq results for NLP7 in root cells overlap significantly with NLP7-bound genes in whole roots[1], N-responsive genes in whole roots[2], and genes directly regulated by nitrate but not by downstream metabolites[3]. The number and percentage of genes in the overlap and the significance of the overlap is indicated for each comparison.

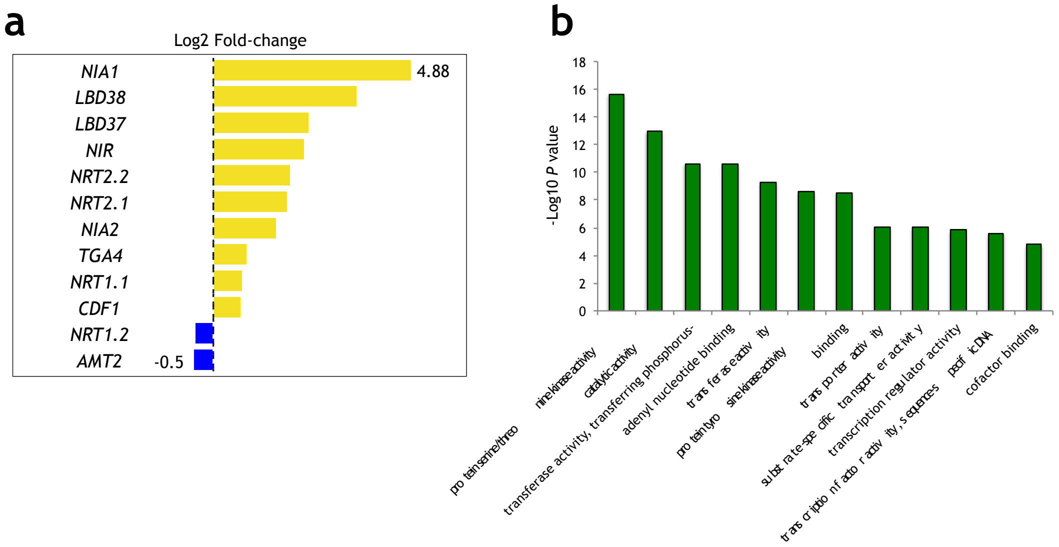

Supplementary Figure 4. Direct NLP7 targets are enriched in GO terms related to transcription factor and kinase activity.

(**a**) Differential expression of direct NLP7 genes known to be involved in N metabolism or transcriptional regulation in response to N. Bars indicate the change of transcript abundance of genes in response to NLP7 nuclear import (+DEX/-DEX, TARGET::+CHX)(Supplementary Data 5). Yellow and blue indicate activation and repression, respectively. (**b**) Gene ontology (GO) terms (molecular function) enriched in the list of NLP7 direct targets.

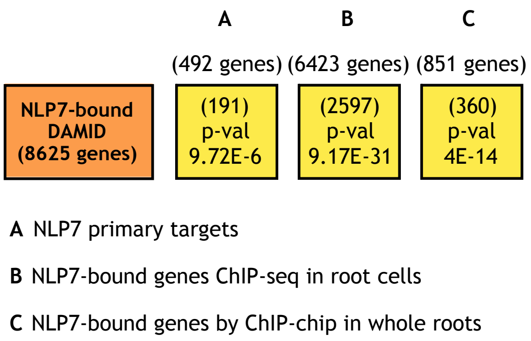

Supplementary Figure 5. DamID-seq of NLP7 captures a significant proportion of NLP7-bound and NLP7 regulated direct targets.

DamID-seq experiment for NLP7 in root cells captures NLP7 directly regulated genes (TARGET::+CHX), NLP7-bound genes in root cells (our study), and NLP7-bound genes in whole roots[1]. Size of overlap is listed in parentheses inside the boxes and significance is indicated for each comparison.

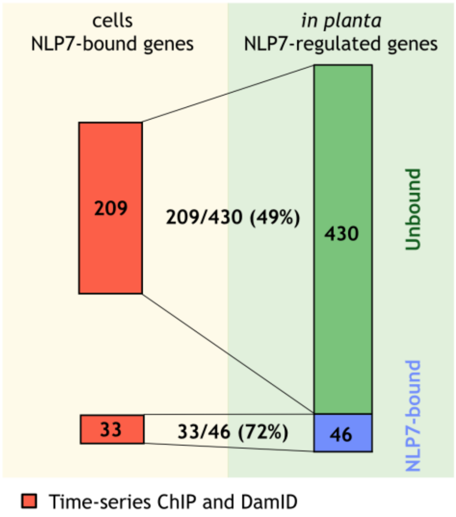

Supplementary Figure 6. Time-series ChIP and DamID in root cells capture transient NLP7 TF-target interactions important for gene regulation in planta but missed using in planta ChIP.

By comparing transcriptome responses of *nlp7-1* mutants to wild-type plants, Marchive *et al.* identified 476 NLP7-regulated genes in whole roots[1]. NLP7 binds only ~10% (46/476) of the NLP7-regulated genes by ChIP assays in planta. By combining time-series ChIP and DamID approaches in cells, we capture 49% (209/430) of the NLP7-regulated but unbound genes in planta. We also capture 72% (33/46) of NLP7-regulated and bound genes in planta.

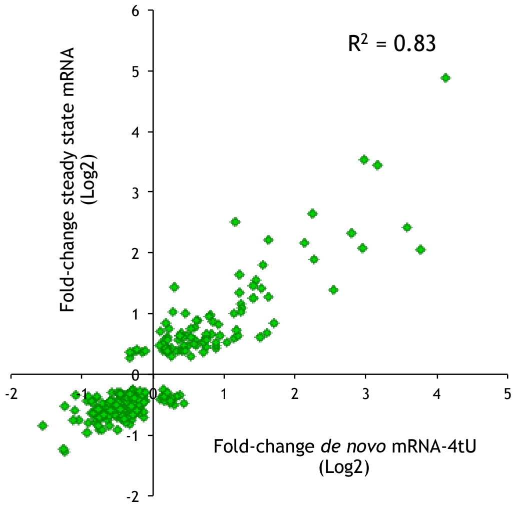

Supplementary Figure 7. Active transcriptional regulation is an important component of changes in target gene transcript levels triggered by NLP7.

The magnitude of steady state mRNA levels (Supplementary Data 5) shows a high degree of correlation with the magnitude of *de novo* mRNA levels captured by 4tU labeling (Supplementary Data 17) for NLP7 direct targets.

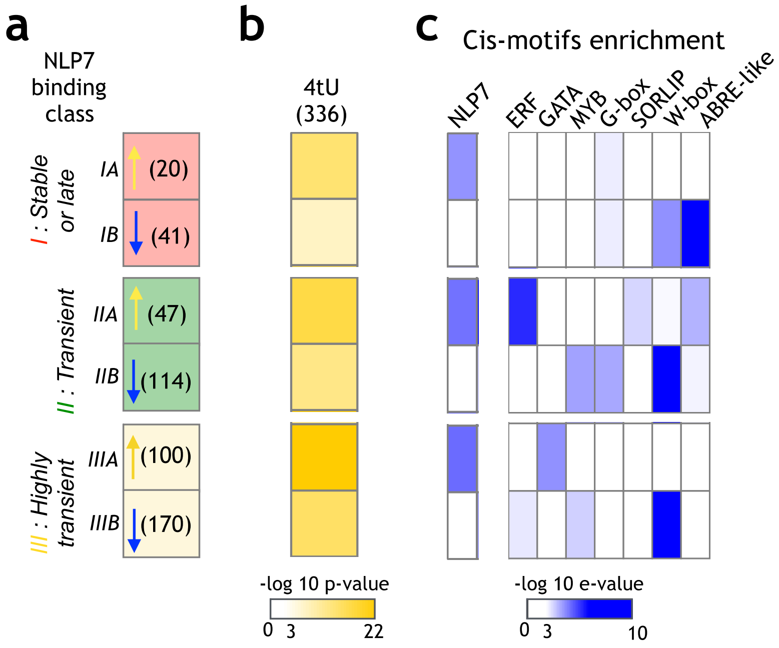

Supplementary Figure 8. The three classes of NLP7 targets are enriched in distinctive cis-motif elements.

(**a**) The three classes were subdivided into induced or repressed (number of genes in parentheses). (**b**) Induced or repressed genes from the three classes are enriched in actively transcribed genes (4tU). (**c**) Class IA, IIA, and IIIA were each enriched in NLP7 cis-motif based on DAP-seq binding[4]. Class IB, IIB and IIIB were not enriched for NLP7 cis-motif but were each enriched in the W-box element. Class IB genes were also highly enriched in ABRE-like elements and class IIB genes were enriched in MYB and G-box elements. No enrichment for additional cis-motifs was found for class IA. In contrast, class IIA, and class IIIA transient targets are specifically enriched in cis-elements for the ERF and GATA families, respectively.

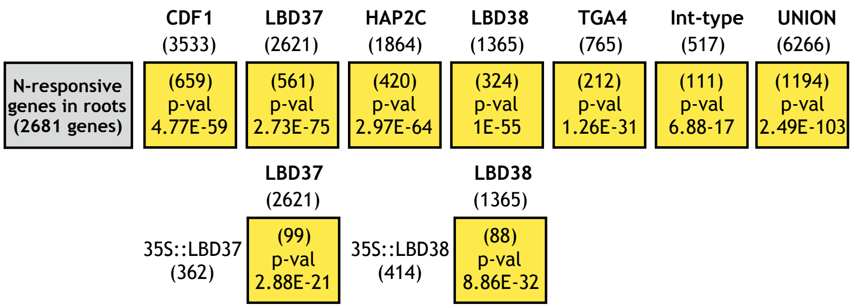

Supplementary Figure 9. Direct targets of secondary TFs identified in root cells have in planta relevance.

Intersection of direct targets of secondary TFs with: (Upper row) N-responsive genes in whole roots[2]; (Lower row) Genes dependent on LBDs detected with overexpression lines (35S::LBD37 and 35S::LBD38) in roots from transgenic plants[5]. Size of overlap is listed in parentheses and significance is indicated for each comparison.

Supplementary References

1. Marchive C*, et al.* Nuclear retention of the transcription factor NLP7 orchestrates the early response to nitrate in plants. *Nature Commun* **4**, 1713 (2013).

2. Varala K*, et al.* Temporal transcriptional logic of dynamic regulatory networks underlying nitrogen signaling and use in plants. *Proc Natl Acad Sci U S A* **115**, 6494-6499 (2018).

3. Wang R*, et al.* Genomic analysis of the nitrate response using a nitrate reductase-null mutant of Arabidopsis. *Plant Physiol* **136**, 2512-2522 (2004).

4. O'Malley RC*, et al.* Cistrome and epicistrome features shape the regulatory DNA landscape. *Cell* **166**, 1598 (2016).

5. Rubin G, Tohge T, Matsuda F, Saito K, Scheible WR. Members of the LBD family of transcription factors repress anthocyanin synthesis and affect additional nitrogen responses in *Arabidopsis*. *Plant Cell* **21**, 3567-3584 (2009).
