# Supplementary material for: Transient genome-wide interactions of the master transcription factor NLP7 initiate a rapid nitrogen-response cascade
Source: Nat Commun. 2020 Mar 2;11:1157. doi: 10.1038/s41467-020-14979-6 (PMC7052136; doi:10.1038/s41467-020-14979-6)
Supplement: Supplementary file 2 — Reporting Summary [file 41467_2020_14979_MOESM2_ESM.pdf]

nature research

Corresponding author(s): Gloria Coruzzi

Last updated by author(s): Jan 27, 2020

Reporting Summary

Nature Research wishes to improve the reproducibility of the work that we publish. This form provides structure for consistency and transparency in reporting. For further information on Nature Research policies, see Authors & Referees and the Editorial Policy Checklist .

Statistics

For all statistical analyses, confirm that the following items are present in the figure legend, table legend, main text, or Methods section.

n/a	Confirmed	
☐	☒	The exact sample size (*n*) for each experimental group/condition, given as a discrete number and unit of measurement
☐	☒	A statement on whether measurements were taken from distinct samples or whether the same sample was measured repeatedly
☐	☒	The statistical test(s) used AND whether they are one- or two-sided<br>*Only common tests should be described solely by name; describe more complex techniques in the Methods section.*
☒	☐	A description of all covariates tested
☐	☒	A description of any assumptions or corrections, such as tests of normality and adjustment for multiple comparisons
☒	☐	A full description of the statistical parameters including central tendency (e.g. means) or other basic estimates (e.g. regression coefficient) AND variation (e.g. standard deviation) or associated estimates of uncertainty (e.g. confidence intervals)
☐	☒	For null hypothesis testing, the test statistic (e.g. *F*, *t*, *r*) with confidence intervals, effect sizes, degrees of freedom and *P* value noted<br>*Give P values as exact values whenever suitable.*
☒	☐	For Bayesian analysis, information on the choice of priors and Markov chain Monte Carlo settings
☒	☐	For hierarchical and complex designs, identification of the appropriate level for tests and full reporting of outcomes
☐	☒	Estimates of effect sizes (e.g. Cohen's *d*, Pearson's *r*), indicating how they were calculated

Our web collection on statistics for biologists contains articles on many of the points above.

Software and code

Policy information about availability of computer code

Data collection

No special software was used for data collection.

Data analysis

>> RNA-seq

RNA-seq reads were mapped to A. thaliana reference genome (Araport 11) using Tophat2 (v2.1.1). Expression levels of all annotated protein-coding genes were estimated by genomicFeatures (v1.34.1) package from Bioconductor. A gene was considered as expressed only its estimated mean of read counts across conditions was > 10. Differentially expressed genes between plus DEX and minus DEX treatments were identified by DESeq2 analysis (Love et al., 2014). Resulting p-values were adjusted for multiple comparisons by false discovery rate (FDR). Differentially regulated genes were then selected with an FDR cutoff of 10 %. We used DREM2 (v2.0.4) to identify patterns of temporal gene expression of the NLP7-dependent cascade.

>> ChIP-seq

The quality of the raw data (FASTQ files) was evaluated by FastQC (http://www.bioinformatics.babraham.ac.uk/projects/fastqc/). Reads were then mapped to the A. thaliana reference genome (Araport 11) using Bowtie2 (version 2.3.2) with parameters "-p 4 -t -x". Redundant reads were removed using Picard tools (v2.8.2). Peak calling was performed using MACS2 (version 2.1.1). Duplicated reads were not considered during peak calling. Peaks were called using the following command: macs2 callpeak -t NLP7_chip_5min.nodup.bam -c input_5min_.nodup.bam -f BAM -g 1.2e8 -n -q 0.05. These peaks were overlapped with the genome annotation (Araport11_GFF3_genes_transposons.201606.gff) to identify genes within 2 kb downstream of the peak using BEDTools (v2.27.1). For visualization purpose, bigwig files were generated by DeepTools (v3.0.1) with the command "bamCoverage"; read coverage was normalized as RPKM (Reads Per Kilobase per Million reads). The matrix to calculate the heatmap was generated using the following options of the computeMatriz tool: computeMatrix reference-point -S nlp7_0min.bw nlp7_5min.bw nlp7_10min.bw nlp7_30min.bw nlp7_180min.bw -R all_peaks.bed --referencePoint center -a 1000 -b 1000 -out result_heatmap. The heatmap was generated using the

plotHeatmap option as follows: plotHeatmap -m result_heatmap -out ./result_pattern_heatmap.pdf --colorMap Purples --sortRegions descend.

>>DamID-seq

Reads obtained from the NLP7-DamID and Dam-only samples were aligned to the A. thaliana reference genome (Araport 11), and the DamID peaks were identified using the same pipeline as for ChIP-seq indicated above. The DamID signal plot was generated by DeepTools (v3.0.1) with the bamCompare tool as follows: bamCompare -b1 nlp7_damid.bam -b2 damid_only.bam -o nlp7_damid_signal.bw -bs 10 -p max --operation log2. The matrix to generate the profile was generated using the computeMatrix tool as follows: computeMatrix scale-regions -S nlp7_damid_signal.bw -R coordinates_damid_bound_genes.bed -a 1000 -b 1000 -o nlp7_damid_signal. The profile was generated as follows: plotProfile -m nlp7_damid_signal -o nlp7_damid_signal.pdf --plotType se --colors orange.

For manuscripts utilizing custom algorithms or software that are central to the research but not yet described in published literature, software must be made available to editors/reviewers. We strongly encourage code deposition in a community repository (e.g. GitHub). See the Nature Research guidelines for submitting code & software for further information.

Data

Policy information about availability of data

All manuscripts must include a data availability statement. This statement should provide the following information, where applicable:

- Accession codes, unique identifiers, or web links for publicly available datasets
- A list of figures that have associated raw data
- A description of any restrictions on data availability

RNA-seq, ChIP-seq and DamID-seq sequencing data is available at the National Center for Biotechnology Information Sequence Read Archive (SRA), with Accession number PRJNA555731. Instructions to download SRA data from NCBI:
https://www.ncbi.nlm.nih.gov/sra/docs/sradownload/#download-sequence-data-files-usi.
Data supporting the findings of this work are available within the paper and its Supplementary Information files.

Field-specific reporting

Please select the one below that is the best fit for your research. If you are not sure, read the appropriate sections before making your selection.

☒ Life sciences ☐ Behavioural & social sciences ☐ Ecological, evolutionary & environmental sciences

For a reference copy of the document with all sections, see nature.com/documents/nr-reporting-summary-flat.pdf

Life sciences study design

All studies must disclose on these points even when the disclosure is negative.

Sample size	For all RNA-seq experiments, we collected ~10,000 transfected protoplasts as one sample, and three independent collections were performed.<br>For ChIP-seq, we collected ~10,000 transfected protoplasts per time point. For DamID-seq, we collected ~10,000 transfected protoplasts for nlp7_DamID and DamID_only experiments.
Data exclusions	No data were excluded from analysis.
Replication	For each RNA-seq experiments, we performed three biological replicates. For the ChIP-seq and DamID-seq experiment, one biological replicate was performed.
Randomization	Plants were grown in a randomized manner and the plates were rotated every three days when the plants were grown in the plant growth incubator.
Blinding	Blinding was not relevant to this study.

Reporting for specific materials, systems and methods

We require information from authors about some types of materials, experimental systems and methods used in many studies. Here, indicate whether each material, system or method listed is relevant to your study. If you are not sure if a list item applies to your research, read the appropriate section before selecting a response.

Materials & experimental systems

n/a	Involved in the study
	[x] Antibodies
[x]	Eukaryotic cell lines
[x]	Palaeontology
[x]	Animals and other organisms
[x]	Human research participants
[x]	Clinical data

Methods

n/a	Involved in the study
	[x] ChIP-seq
[x]	Flow cytometry
[x]	MRI-based neuroimaging

Antibodies

Antibodies used	Antibodies used in this study are commercially available: anti-GR antibody (GR P-20, Santa Cruz biotech).
Validation	The specificity of the antibodies had been tested by the supplier.

ChIP-seq

Data deposition

[x] Confirm that both raw and final processed data have been deposited in a public database such as GEO.

[x] Confirm that you have deposited or provided access to graph files (e.g. BED files) for the called peaks.

Data access links<br>*May remain private before publication.*	https://www.ncbi.nlm.nih.gov/bioproject/PRJNA555731.<br>Instructions to download Sequence Read Archive (SRA) from NCBI:<br>https://www.ncbi.nlm.nih.gov/sra/docs/sradownload/#download-sequence-data-files-usi.
Files in database submission	C7D4YACXX_l01n01_nlp7_chip_0_pchx_pn.fastq.gz<br>C7D4YACXX_l01n01_nlp7_chip_5_pchx_pn.fastq.gz<br>C7D4YACXX_l01n01_nlp7_chip_10_pchx_pn.fastq.gz<br>C7D4YACXX_l01n01_nlp7_chip_30_pchx_pn.fastq.gz<br>C7D4YACXX_l01n01_nlp7_chip_180_pchx_pn.fastq.gz<br>C7D4YACXX_l01n01_nlp7_chip_input_0_pchx_pn.fastq.gz<br>C7D4YACXX_l01n01_nlp7_chip_input_5_pchx_pn.fastq.gz<br>C7D4YACXX_l01n01_nlp7_chip_input_10_pchx_pn.fastq.gz<br>C7D4YACXX_l01n01_nlp7_chip_input_30_pchx_pn.fastq.gz<br>C7D4YACXX_l01n01_nlp7_chip_input_180_pchx_pn.fastq.gz
Genome browser session<br>(e.g. UCSC)	NA

Methodology

Replicates	NA
Sequencing depth	The average unique mapped read number for the ChIP samples is ca. 3.6M. Read length: 75bp in single-end.
Antibodies	anti-GR antibody (GR P-20, Santa Cruz biotech) cat:sc-1002. Lot # L1212.
Peak calling parameters	Peak calling was performed using MACS2 (version 2.1.1). Duplicated reads were not considered during peak calling. Peaks were called using the following command: macs2 callpeak -t NLP7_chip_5min.nodup.bam -c input_5min_.nodup.bam -f BAM -g 1.2e8 -n -q 0.05.
Data quality	We calculated the fraction of reads in peaks (FRiP) as a measure of ChIP-seq quality. We obtained a FRiP score >3%. According to the ENCODE consortia, a FRiP enrichment of 1% or more when peaks are called using MACS2 with default parameters is considered as acceptable (https://www.ncbi.nlm.nih.gov/pmc/articles/PMC3431496/). In addition, a high proportion of NLP7 peaks (~90%) are located within -2 kb and the transcription start site (TSS) of genes supporting the high quality of our data.
Software	The quality of the raw data (FASTQ files) was evaluated by FastQC (http://www.bioinformatics.babraham.ac.uk/projects/fastqc/). FRiP score for each ChIP-sample was calculated with DeepTools (https://deeptools.readthedocs.io/en/develop/content/example_api_tutorial.html#computing-the-frip-score). ChIP-seq data signal tracks were visualized in the Integrative Genomics Viewer (IGV) (https://software.broadinstitute.org/software/igv/).
