# Supplementary material for: Transient genome-wide interactions of the master transcription factor NLP7 initiate a rapid nitrogen-response cascade
Source: Nat Commun. 2020 Mar 2;11:1157. doi: 10.1038/s41467-020-14979-6 (PMC7052136; doi:10.1038/s41467-020-14979-6)
Supplement: Supplementary file 3 — Description of Additional Supplementary Files [file 41467_2020_14979_MOESM3_ESM.docx]

**Description of Additional Supplementary Files**

File Name: Supplementary Data 1

Description: NLP7 ChIP-seq peaks and genes 5 minutes

File Name: Supplementary Data 2

Description: NLP7 ChIP-seq peaks and genes 10 minutes

File Name: Supplementary Data 3

Description: NLP7 ChIP-seq peaks and genes 30 minutes

File Name: Supplementary Data 4

Description: NLP7 ChIP-seq peaks and genes 180 minutes

File Name: Supplementary Data 5

Description: NLP7 directly regulated genes

File Name: Supplementary Data 6

Description: Transcription Factors regulated by NLP7

File Name: Supplementary Data 7

Description: Kinases regulated by NLP7

File Name: Supplementary Data 7

Description: Kinases regulated by NLP7

File Name: Supplementary Data 8

Description: Class I: Stable or late genes

File Name: Supplementary Data 9

Description: Class II: Transient genes

File Name: Supplementary Data 10

Description: Class III: Highly transient

File Name: Supplementary Data 11

Description: NLP7 DamID-seq peaks and genes

File Name: Supplementary Data 12

Description: NLP7 direct targets captured by DamID-seq

File Name: Supplementary Data 13

Description: Genes captured by DamID-seq that were missed by ChIP-seq

File Name: Supplementary Data 14

Description: NLP7-regulated-not detectably bound genes in planta and captured as NLP7-bound in root cells

File Name: Supplementary Data 15

Description: NLP7 binding motif in the promoter of genes captured by DamID

File Name: Supplementary Data 16

Description: NLP7 binding motif in the promoter of genes captured by ChIP

File Name: Supplementary Data 17

Description: Actively transcribed NLP7 targets captured by 4tU labelling

File Name: Supplementary Data 18

Description: NLP7 indirect targets

File Name: Supplementary Data 19

Description: Targets of CDF1 (At5g62430)

File Name: Supplementary Data 20

Description: Targets of LBD37 (At5g67420)

File Name: Supplementary Data 21

Description: Targets of HAP2C (At1g72830)

File Name: Supplementary Data 22

Description: Targets of LBD38 (At3g49940)

File Name: Supplementary Data 23

Description: Targets of TGA4 (At5g10030)

File Name: Supplementary Data 24

Description: Targets of Integrase-Type DNA (At4g39780)

File Name: Supplementary Data 25

Description: Targets of NAC096 (At5g46590)

File Name: Supplementary Data 26

Description: Gene Ontology terms enriched in the N-regulated genes controlled by TF2s

File Name: Supplementary Data 27

Description: N-regulated genes in the NLP7-dependent transcriptional cascade

File Name: Supplementary Data 28

Description: Coding sequence of NLP7 depleted in GATC sites compatible with pDamBOB vector
